# Decreased Exosomal Acetylcholinesterase Activity in the Plasma of Patients With Parkinson’s Disease

**DOI:** 10.3389/fnagi.2021.665400

**Published:** 2021-05-28

**Authors:** Kyu Hwan Shim, Han Gyeol Go, Heewon Bae, Da-Eun Jeong, Danyeong Kim, Young Chul Youn, SangYun Kim, Seong Soo A. An, Min Ju Kang

**Affiliations:** ^1^Department of Neurology, Veterans Medical Research Institute, Veterans Health Service Medical Center, Seoul, South Korea; ^2^Department of Bionano Technology, Gachon University, Seongnam-si, South Korea; ^3^Department of Neurology, Chung-Ang University Hospital, Seoul, South Korea; ^4^Department of Neurology, Seoul National University Bundang Hospital and Seoul National University College of Medicine, Seongnam-si, South Korea

**Keywords:** Parkinson’s disease, acetylcholinesterase, exosome, plasma, ultracentrifugation, alpha-synuclein

## Abstract

Exosomes, which are small extracellular vesicles produced from various cell types, contain a variety of molecular constituents, such as proteins, lipids, and RNA. Recently, exosomal biomarkers have been investigated to probe the understanding and diagnosis of neurodegenerative disorders. Previous reports have demonstrated increased exosomal α-synuclein (α-syn) in patients with Parkinson’s disease (PD) in comparison to healthy controls (HC). Interestingly, the cholinergic loss was revealed in the central and peripheral nervous systems in histopathology and molecular neuroimaging. Thereby, we simultaneously examined acetylcholinesterase (AChE) with α-syn as exosomal markers. Exosomes were isolated from the plasma of 34 FP-CIT PET proven patients with PD and 29 HC. Exosomal α-syn and AChE activity were quantified andthe relationship with clinical parameters was analyzed. Remarkably, exosomal AChE activity was significantly decreased in PD compared to HC (*P* = 0.002). Moreover, exosomal AChE activity in PD revealed a strong negative correlation with disease severity, including H&Y (*P* = 0.007) and UPDRS part III (*P* = 0.047) scores. By contrast, no significant difference in exosomal α-syn concentration was observed between groups. These results support the occurrence of cholinergic dysfunction in PD, and they could be implicated with disease progression, especially motor deficits. Exosomal AChE activity with advanced exosome isolation techniques may be a reliable biomarker for the early diagnosis and prognosis of PD.

## Introduction

One of the pathophysiological characteristics of Parkinson’s disease (PD) is the formation of Lewy bodies in the brain (Spillantini et al., 1997), which are mainly composed of insoluble aggregated α-synuclein (α-syn; Shults, 2006). Accordingly, α-syn has been studied extensively as a potential biomarker and indicator of disease progression in PD and its related synucleinopathies (El-Agnaf et al., 2003; Hong et al., 2010; Devic et al., 2011; Mollenhauer et al., 2011; Shi et al., 2011). However, inconsistent plasma α-syn data in PD have been reported, and the diagnostic performance of α-syn in biofluids was still insufficient for application in PD diagnosis (Lee et al., 2006; Li et al., 2007; Foulds et al., 2011, 2013; Gorostidi et al., 2012; Ishii et al., 2015; Lin et al., 2017; Shim et al., 2020). The absence of reliable biofluidic biomarkers for PD has limited the monitoring of disease progression or treatment response.

Recent exosome studies have presented diverse possibilities for the etiology, diagnosis, and treatment of previously unexplained diseases (Lin et al., 2015). Exosomes, which are small extracellular vesicles with proteins and other components, are communication vehicles between cells or tissues. The mechanism of α-syn secretion via exosomes is not fully understood; however, several studies have demonstrated the secretion of α-syn on membrane vesicles of endocytic origin, sized 30–150 nm (Lee et al., 2005; Emmanouilidou et al., 2010; Alvarez-Erviti et al., 2011; Danzer et al., 2012; Tofaris, 2017). Recently, researchers have attempted to use exosomes in diagnosing PD by isolating them from their biofluids (Ho et al., 2014; Shi et al., 2014; Wu et al., 2017; Cerri et al., 2018; Zhao et al., 2019; Jiang et al., 2020; Niu et al., 2020). In particular, the levels of exosomal α-syn isolated from the blood were higher in the patients with PD than in the control group (Shi et al., 2014; Cerri et al., 2018; Zhao et al., 2019; Jiang et al., 2020). Accumulated results are required for explicit interpretations; therefore, detecting exosomal biomarkers from biofluids may contribute to the discovery of other potential PD biomarkers.

PD is a multifaceted and complex disorder, affecting multiple neurotransmissions rather than a single-system neurodegenerative disease (Braak et al., 2003; Langston, 2006). Growing evidence has suggested an association between degeneration of the nigrostriatal dopaminergic system and cholinergic denervation in PD (Müller and Bohnen, 2013). Interestingly, a study using positron emission tomography (PET), which measured acetylcholinesterase (AChE) activity, has reported severe cholinergic denervation in the brain of patients with PD when compared with Alzheimer’s disease (AD; Bohnen et al., 2003). Moreover, 11C-donepezil uptake is significantly reduced in the peripheral organs in PD patients, indicating a decrease in AChE in the peripheral system (Gjerløff et al., 2014; Fedorova et al., 2017). Taken together, this suggests that AChE is extensively associated with the pathophysiological process of PD in the peripheral organs and the brain.

In the present study, exosomes were isolated from the plasma samples of patients with PD and healthy controls (HC) using ultracentrifugation. Exosomal AChE activity and α-syn levels were measured to investigate the correlation with clinical parameters and their biomarker potential.

## Materials and Methods

### Participants

Patients with PD were recruited between May 2019 and June 2020 from the Veterans Health Service Medical Center. PD was diagnosed based on the UK Parkinson’s Disease Society Brain Bank criteria. All patients underwent FP-CIT PET, were staged according to Hoehn and Yahr (H&Y) criteria, and evaluated by the Unified Parkinson’s Disease Rating Scale (UPDRS). Age-matched HC who had no evidence of neurological or serious medical illnesses in their medical history or via neurological examination were recruited. All participants underwent brain magnetic resonance imaging and Mini-Mental State Examination (MMSE) to evaluate global cognitive impairments. All participants or their legal representatives provided written informed consent. The study protocol was approved by the Institutional Review Board of Veterans Healthcare Medical Center (2019-05-004).

### Sampling

Blood samples were collected in tubes containing EDTA to prevent blood clotting. Plasma was separated within 4 h after blood collection. Samples were centrifuged for 10 min at 1,500 *g* to eliminate red blood cells, platelets, and cell debris. The separated plasma was transferred to the polypropylene tube and stored at −80°C until analysis.

### Plasma Exosome Isolation

Plasma (1 ml) was centrifuged at 20,000 *g* for 1 h to remove large extracellular vesicles. The supernatant was transferred in an ultra-centrifugation tube filled with phosphate-buffered saline (PBS; 28348, Thermo Fisher) and ultra-centrifugated at 100,000 *g* (Optima XE-90 with 90Ti rotor, Beckman Coulter) for 1.5 h to pellet exosomes. The supernatant was discarded, and the pellets were resuspended in PBS with phosphate (4906845001, Roche) and protease (5892970001, Roche) inhibitors. Exosomes were lysed by incubating with M-PER (Cat. #78501; Thermo Fisher Scientific, Carlsbad, CA, USA), a mild detergent lysis buffer that yields highly efficient soluble proteins in a non-denatured state, for 30 min at 4°C (Guix et al., 2018). BCA assay (23227, Thermo Fisher Scientific, USA) was performed to quantify the exosomal protein concentration, according to the manufacturer’s instructions. The plates were read using a plate reader (SpectraMax Plus 384; Molecular Devices, Sunnyvale, CA, USA) at 540 nm.

### Transmission Electron Microscopy

The exosomes were fixed with 4% formaldehyde in PBS for 1 h at 4°C. Fixed samples were dropped onto formvar carbon film-coated 150 mesh copper grids for 1 min. The filter paper was used to remove any excess sample. The grids were negatively stained with 2% uranyl acetate. The excess liquid was washed using filter paper, and imaging was performed on a Hitachi H7600 transmission electron microscope, operated at 80 kV.

### Nanoparticle Tracking Analysis

Nanoparticle tracking analysis was conducted with the isolated plasma exosomes to determine the concentration and the size distribution of particles using a ZetaView (Particle Metrix). The samples were diluted properly with particle-free PBS. Videos of the light-refracting particles were recorded with the following settings: 25°C fixed temperature, 11 positions, three cycles, sensitivity 80, shutter 100, 30 fps, 5 s videos/position, three measurements. The number and size distribution were analyzed by ZetaView Analyze 08.05.12. SP2.

### Western Blot Analysis

The isolated exosomes (~20 μg proteins) were solubilized with Laemmli sample buffer and heated for 5 min at 98°C. The proteins were separated on an SDS-PAGE gel and transferred to a PVDF membrane. The membranes were blocked with 3% Nonfat-dried milk bovine (Sigma, M7409) in TBST (Thermo, #28360) for 1 h at room temperature (RT). Primary antibodies against Alix (Cell Signalling Technology, #2171) and GM130 (Cell signaling Technology, #12480) were diluted 1:1,000 in TBST with 3% Nonfat-dried milk bovine and incubated overnight at 4°C. The membranes were washed with TBST and incubated with HRP-conjugated secondary antibodies for 1 h at RT. The immunostained proteins were detected by applying chemiluminescent substrate (Thermo, #34577) and imaged by the DAVINCH-Chemi imager.

### Exosomal Acetylcholinesterase Activity Measurement

AChE activity was determined using a fluorometric acetylcholinesterase assay kit (ab138872, Abcam). The following procedure was performed according to the manufacturer’s instructions. Briefly, AChE standard and samples were diluted in assay buffer and applied to a 96-well plate in duplicate. The reaction mixture was added to each well and incubated for 1 h at RT. The fluorescence signal was monitored using a fluorescence microplate reader (FLUOstar Omega; BMG Labtech Inc., Cary, NC, USA; λex. = 490 nm, λem. = 520 nm).

### Quantification of Exosomal α-Synuclein

Exosomal α-syn was measured by a commercial ELISA kit (AS-55550-H, Anaspec) according to the manufacturer’s instructions. Briefly, exosomal lysates were diluted in sample buffer and added to each well in duplicate. After 4 h of incubation at RT, the microplate was washed six times, then TMB was applied to each well. Stop solution was added to terminate the reaction, and the optical signal was determined at 450 nm using a microplate reader.

### Statistical Analysis

Statistical analyses were conducted using SPSS 24 (SPSS Inc., Chicago, IL). Fisher’s exact test was used to assess the sex difference. Student’s *t*- and Mann-Whitney *U* tests were performed to compare the biomarker between groups. *P* < 0.05 was regarded as statistically significant. Significant correlations were assessed using Spearman’s correlation coefficient. The receiver operating characteristic (ROC) curve was analyzed to assess the accuracy of the diagnostic performance by calculating the area under the curve (AUC). The cutoff value was determined when the sum of the sensitivity and specificity maximized the Youden index.

## Results

### Participant Demographics and Clinical Characteristics

Demographic characteristics and clinical features are summarized in Table 1. No significant differences in age or sex were found between groups; however, males were recruited more than females due to the specificity of recruitment from a veteran’s hospital. There was no difference in MMSE score between groups. FP-CIT PET was performed in all patients with PD; all patients presented with reduced dopamine transport activity. In the HC group, seven individuals underwent FP-CIT PET; this revealed normal dopamine transport activity.

**Table 1:** Demographics and clinical characteristics of the Parkinson’s disease (PD) and healthy controls (HC) groups.

	PD	HC	*P*-value
*N* of individuals	34	29	-
Age	74.2 (4.7)	73.9 (4.6)	0.816^a^
Sex (M/F)	30/4	24/5	0.721^b^
MMSE	24.7 (3.8)	25.8 (2.5)	0.232^c^
H&Y score (1/1.5/2/2.5)	15/8/6/5	-	-
UPDRS part III	40.0 (13.1)	-	-
Disease duration (year)	5.1 (4.3)	-	-
FP-CIT PET (P/N)	34/0	0/7	0.000^b^

*Data are presented as mean (standard deviation); ^a^Student’s *t*-test; ^b^Fisher’s Exact test; ^c^Mann-Whitney test*.

### Analysis of Plasma Exosomal Biomarkers in Clinical Samples

**Figure 1:**
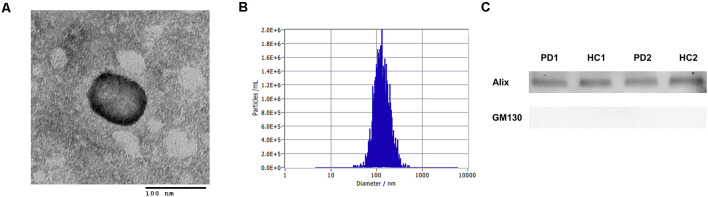
Characteristics of the isolated exosomes by ultracentrifugation. **(A)** Representative transmission electron micrograph of isolated plasma exosomes (Scale bar = 100 nm). **(B)** A representative plot depicting the size and concentration of exosomes. **(C)** Western blot of the exosomes from Parkinson’s disease (PD) and healthy controls (HC) individuals with specific antibodies against Alix and GM130 as positive and negative controls, respectively.

**Figure 2:**
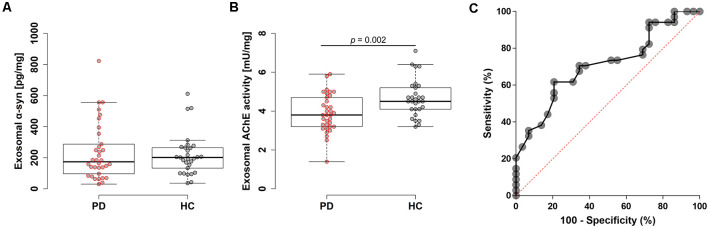
Evaluation of plasma exosomal biomarkers. Box plots of exosomal α-syn levels **(A)** and acetylcholinesterase (AChE) activity **(B)**. Boxes represent the interquartile range; the horizontal lines mean medians and the whiskers. The Student’s *t*-test was used for comparison of the two groups. **(C)** Receiver operating characteristic (ROC) analysis of exosomal AChE activity.

Diagnostic performances were evaluated by measuring AChE activity and α-syn levels from the isolated plasma exosomes. Exosomes were isolated via ultracentrifugation, which was confirmed by visualization with transmission electron microscopy and nanoparticle tracking analysis (Figure 1A and Supplementary Figure 1C). The estimated size of exosomes was distributed around 140 nm by nanoparticle tracking analysis (Figure 1B). In Western blot, exosomes were enriched in Alix, but not in GM130 (Figure 1C). No differences in plasma exosome size and concentration were found between PD and HC (Supplementary Figures 1A,B). The total amount of proteins in the isolated exosomes may vary between individuals; therefore, total exosomal protein content was measured and used to normalize the two biomarkers. Total exosomal protein levels were highly correlated with the concentration of exosomes and the exosomal AChE activity, indicating the adequacy as a normalization factor (Supplementary Figure 2). The concentrations of exosomal α-syn showed no difference between groups (PD, 234.0 ± 183.2 pg/mg; HC, 219.7 ± 135.3 pg/mg; Figure 2A). Remarkably, exosomal AChE activity was significantly decreased in PD group (3.9 ± 1.0 mU/mg) when compared with HC group (4.7 ± 1.0 mU/mg; *P* = 0.002; Figure 2B). The ROC curve analysis revealed that exosomal AChE activity exhibited a moderate performance for PD diagnosis (AUC = 0.709; 95% confidence interval, 0.582–0.836; Figure 2C). When a cutoff value of 4.05 was applied, sensitivity and specificity were 61.8% and 79.3%, respectively.

### Correlation Between Exosomal AChE Activity and PD Progression

**Figure 3:**
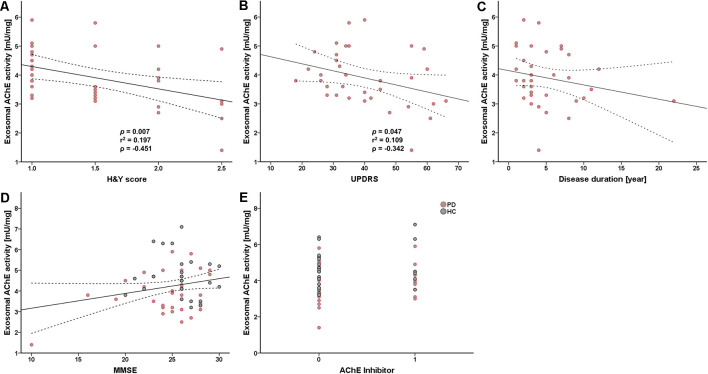
Scatter plot of exosomal AChE activity and clinical data. Correlation between AChE activity and H&Y scores **(A)**, UPDRS part III score **(B)**, disease duration **(C)**, Mini-mental state examination (MMSE) score **(D)**, and AChE inhibitor use (donepezil, galantamine, and rivastigmine) **(E)**. 0 = without AChE inhibitors; 1 = with AChE inhibitors. The dashed line represents the 95% prediction interval of the regression line. ρ, Spearman’s rho. Spearman’s correlation analysis was used to determine any statistical significance.

Next, we analyzed the correlation between exosomal AChE activity and disease severity in patients with PD to investigate its clinical implication. Intriguingly, exosomal AChE activity was significantly correlated with H&Y (*p* = 0.007, *r^2^* = 0.197, *ρ* = −0.451) and UPDRS part III scores (*p* = 0.047, *r^2^* = 0.109, *ρ* = −0.342; Figures 3A,B). Disease duration tended towards a moderate negative correlation with exosomal AChE activity (*ρ* = 0.101; Figure 3C). MMSE score and the administration of AChE inhibitors, including donepezil, galantamine, and rivastigmine, were not significantly correlated with exosomal AChE activity (Figures 3D,E).

## Discussion

In the current study, exosomal AChE activity was significantly reduced in the PD group compared to the HC group. Moreover, exosomal AChE activity was significantly negatively correlated with disease severity. Previous studies have examined the cholinergic deficits in PD via histopathology and molecular neuroimaging. A reduced number of cholinergic neurons was reported in the pedunculopontine nuclei of PD patients, which was correlated with H&Y scores (Rinne et al., 2008). A cholinergic PET study showed that patients of PD and PD with dementia had cholinergic denervation in the medial secondary occipital cortex (Müller and Bohnen, 2013). Additionally, the cholinergic loss was found using 11C-donepezil PET, which supported parasympathetic denervation in the peripheral nervous system of PD patients (Gjerløff et al., 2014; Fedorova et al., 2017). A significant decrease in colonic 11C-donepezil signal in the early stages of PD might explain the increased constipation as the first nonmotor symptom in the prodromal phase (Fedorova et al., 2017). Taken together, these studies demonstrate cholinergic dysfunction in the central and peripheral nervous systems of patients with PD, which supports the reduction in exosomal AChE activity found in this study. Furthermore, the strong association between the decreased exosomal AChE activity and disease severity highlights the importance of AChE in PD pathophysiology. Exosomal AChE activity was an insufficient single diagnostic biomarker for PD; however, it may have the potential to further elucidate PD pathophysiology. Despite the exosomal AChE levels had no difference between the two groups in the preliminary results (Supplementary Figure 3), we will keep considering the measurement of exosomal AChE levels to improve the diagnostic performance in the future.

The etiology of the decreased AChE activity in PD remains obscure. One hypothesis is that the decreased production and secretion of exosomal AChE are associated with α-syn-induced neuronal death. Moreover, α-syn aggregates interfere with the axonal transport of protein (Chung et al., 2009; Chu et al., 2012); therefore, exosomal AChE activity might be decreased via related mechanisms. A different hypothesis relating to the balance between acetylcholine (ACh) and dopamine (DA) may explain this phenomenon (Spehlmann and Stahl, 1976; Aosaki et al., 2010). This balance is the main feature of motor activity control. DA deficiency in PD pathology can trigger a severe decline in basal ganglia circuit dynamics, leading to motor and cognitive impairment with cholinergic system imbalance (Aosaki et al., 2010). In a mouse PD model genetically modified for a decline in DA levels, an imbalance between ACh and DA exacerbated motor deficit (McKinley et al., 2019). Interestingly, administration of a high dose of donepezil highlighted that disturbing the balance of ACh and DA leads to medication-induced Parkinsonism (Rangseekajee et al., 2016). In the current study, it could be hypothesized that an imbalance between DA and ACh via DA depletion exacerbated the decrease in AChE activity.

In previous studies, exosomal α-syn was increased in PD when compared with HC (Shi et al., 2014; Cerri et al., 2018; Zhao et al., 2019; Jiang et al., 2020; Niu et al., 2020). By contrast, other studies have shown a decrease (Stuendl et al., 2016; Si et al., 2019) or no difference in exosomal α-syn between PD and HC groups (Tomlinson et al., 2015; Cao et al., 2019). These discrepancies may be due to biofluid type or technical differences in exosome isolation. For example, ultracentrifugation, as performed in this study, resulted in total exosome isolation from plasma; however, L1CAM antibody could specifically separate neuronal-derived exosomes in other studies. Subsequently, results may differ based on exosome origin, which suggests different pathways and significance. Therefore, comparing several exosome isolation techniques and subdividing them into specific cell-derived exosomes would best elucidate these mechanisms.

There are several limitations in the current study. First, the cohort was recruited from a limited number of individuals; therefore, the current study should be extended and reproduced with a larger cohort. Second, we recruited more male than female patients in a single center; therefore, the next study should recruit the samples from a multicenter cohort. Third, no organ-specific derived exosomes were extracted. Exosomes are released from various cells, including neurons, blood cells, and epithelial cells; therefore, priority should be given to isolating neuron-derived exosomes to investigate AChE activity in subsequent studies.

## Conclusion

This study demonstrated the reduced AChE activity in plasma exosomes from patients with PD when compared with HC. Furthermore, a significant negative correlation between AChE activity and disease severity was observed in the PD group. These results suggested that plasma exosomal AChE could serve as a surrogate biomarker to monitor disease progression. Exosomal AChE activity showed a moderate diagnostic performance, which could be improved by applying an advanced exosome isolation technique. Exosomes may play pivotal roles in the occurrence and progression of PD; therefore, further studies will continue to elucidate the pathophysiology of PD and develop diagnostic and therapeutic tools.

## Data Availability Statement

The raw data supporting the conclusions of this article will be made available by the authors, without undue reservation.

## Ethics Statement

The studies involving human participants were reviewed and approved by Institutional Review Board of Veterans Healthcare Medical Center. The patients/participants provided their written informed consent to participate in this study.

## Author Contributions

KS, SA, and MK: conceptualization. HB: data curation. MK: funding acquisition. KS, HG, and DK: investigation. KS and MK: project administration. D-EJ and MK: resources. YY, SK, and SA: supervision. SA and MK: validation. KS and MK: writing—original draft. SA: writing—review and editing. All authors contributed to the article and approved the submitted version.

## Conflict of Interest

The authors declare that the research was conducted in the absence of any commercial or financial relationships that could be construed as a potential conflict of interest.
