# Corrigendum: Decreased Exosomal Acetylcholinesterase Activity in the Plasma of Patients With Parkinson's Disease

**DOI:** 10.3389/fnagi.2021.726525

**Published:** 2021-07-07

**Authors:** Kyu Hwan Shim, Han Gyeol Go, Heewon Bae, Da-Eun Jeong, Danyeong Kim, Young Chul Youn, SangYun Kim, Seong Soo A. An, Min Ju Kang

**Affiliations:** ^1^Department of Neurology, Veterans Medical Research Institute, Veterans Health Service Medical Center, Seoul, South Korea; ^2^Department of Bionano Technology, Gachon University, Seongnam-si, South Korea; ^3^Department of Neurology, Chung-Ang University Hospital, Seoul, South Korea; ^4^Department of Neurology, Seoul National University Bundang Hospital and Seoul National University College of Medicine, Seongnam-si, South Korea

**Keywords:** Parkinson's disease, acetylcholinesterase, exosome, plasma, ultracentrifugation, alpha-synuclein

In the original article, we neglected to include the funder National Research Foundation of Korea, NRF-2020R1A2B5B01002463 to Seong Soo A. An.

The authors apologize for this error and state that this does not change the scientific conclusions of the article in any way. The original article has been updated.

