# Supplementary material for: Decreased Exosomal Acetylcholinesterase Activity in the Plasma of Patients With Parkinson’s Disease
Source: Front Aging Neurosci. 2021 May 28;13:665400. doi: 10.3389/fnagi.2021.665400 (PMC8193230; doi:10.3389/fnagi.2021.665400)
Supplement: Supplementary file 1 [file Data_Sheet_1.DOCX]

Supplementary Materials and Methods

1. **Exosomal acetylcholinesterase level measurement**

Exosomal acetylcholinesterase levels were quantified by a commercial ELISA kit (DY7574-05, R&D Systems), according to the manufacturer’s instructions. Exosomal lysates (15 μL) were added into each well with the reaction diluent (85 μL) and incubated for 2 hours at room temperature. A plate was washed three times with 400 μL of wash buffer at each step. The detection antibody was applied and incubated 2 hours at room temperature. After incubation with HRP conjugated streptavidin for 20 minutes, TMB solution was inserted for color development by HRP. The reaction was stopped by adding the stop solution, and the signal was recorded at 450 nm using a microplate reader.

Supplementary Results


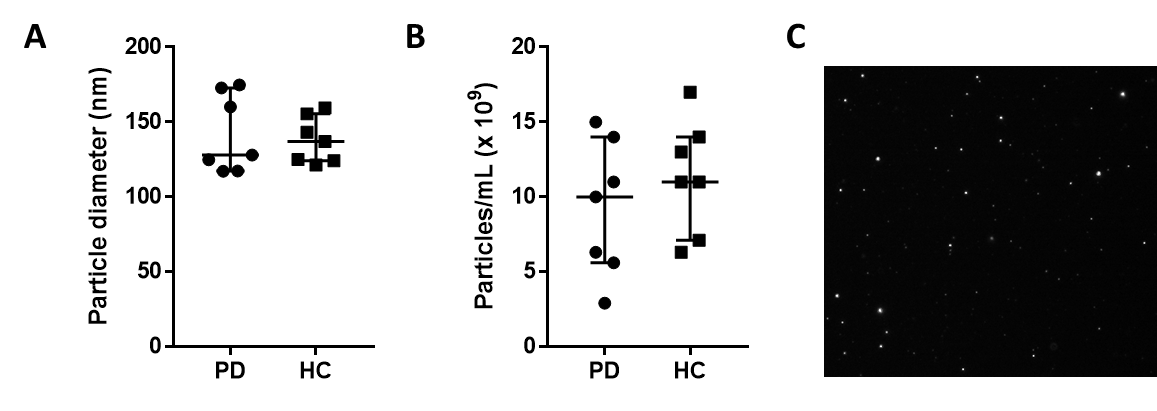


**Supplementary Figure 1.** Characterization of plasma exosomes. The median size **(A)** and the median concentration **(B)** of exosomes in PD and HC. Solid horizontal lines and error bars represent median and interquartile range, respectively. **(C)** A representative image of exosomes.


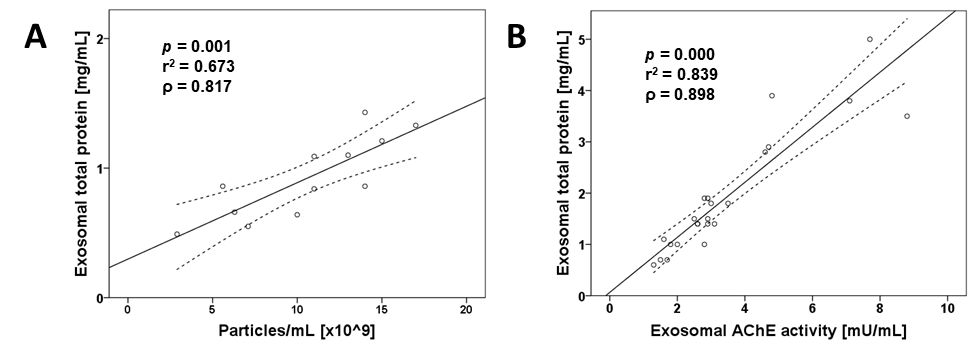


**Supplementary Figure 2.** Association of the exosomal total protein levels with the concentration of exosomes **(A)** and the exosomal AChE activity **(B)**. The dashed line represents the 95% prediction interval of the regression line. ρ, Spearman’s rho. Spearman’s correlation analysis was used to determine any statistical significance.


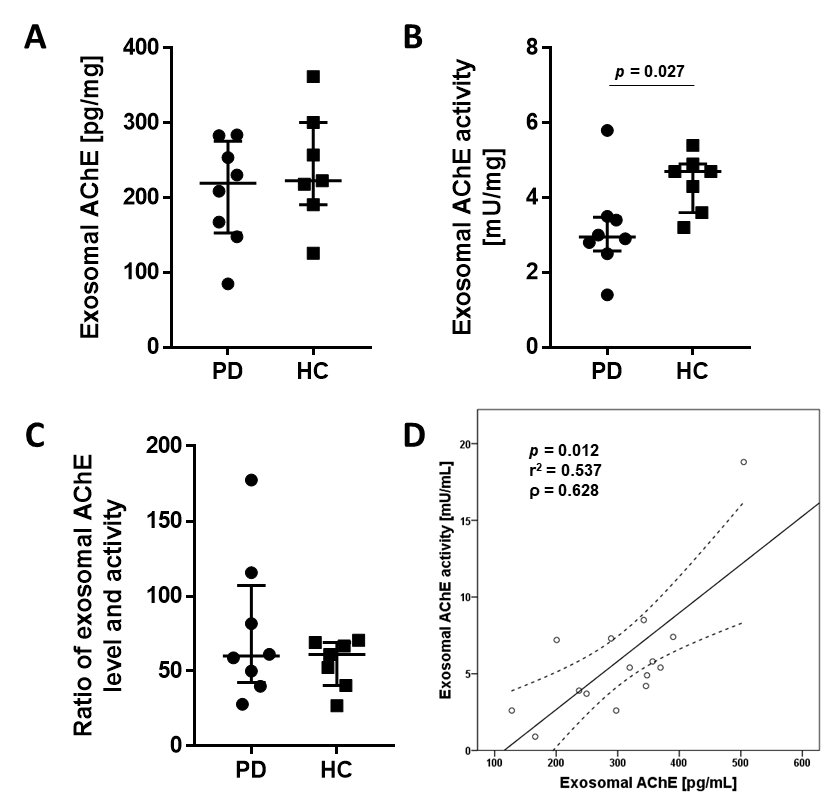


**Supplementary Figure 3.** Measurement of AChE levels and activity in the exosomes. Scatter plot of the exosomal AChE levels (**A**), activity (**B**), and their ratio (**C**) in PD and HC. Mann-Whitney test was used for comparison of the two groups. Solid horizontal lines and error bars represent median and interquartile range, respectively. (**D**) Correlation between the exosomal AChE levels and activity. The dashed line represents the 95% prediction interval of the regression line. ρ, Spearman’s rho. Spearman’s correlation analysis was used to determine any statistical significance.
